# Towards peak experience - exploring the influence of wilderness on conscious awareness

**DOI:** 10.1192/bjo.2021.748

**Published:** 2021-06-18

**Authors:** Oli Purnell

**Affiliations:** Swansea University Medical School

## Abstract

This study examines what effect wilderness has on our conscious awareness, and by extension of that meta-cognition; our physical, mental and spiritual health. It reviews available scientific and artistic literature and integrates this with interviews in order to generate original grounded theory. It was found that intensity of wilderness experience varied proportionally with four mediators; Challenge, Immersion, Beauty and Time. With these maximised, experiences broadly within four themes occurred; Increased Awareness, Confidence, Perspective and Connectedness. When sufficiently intense, these four experiences amalgamated to elicit what Maslow described as 'Peak Experience'. As such, this thesis unexpectedly provides a pragmatic recipie towards peak experience, and a map of one's potential psychological journey, in the context of wilderness.

